# InAs/GaAs Quantum Dot Microlasers Formed on Silicon Using Monolithic and Hybrid Integration Methods

**DOI:** 10.3390/ma13102315

**Published:** 2020-05-18

**Authors:** Alexey E. Zhukov, Natalia V. Kryzhanovskaya, Eduard I. Moiseev, Anna S. Dragunova, Mingchu Tang, Siming Chen, Huiyun Liu, Marina M. Kulagina, Svetlana A. Kadinskaya, Fedor I. Zubov, Alexey M. Mozharov, Mikhail V. Maximov

**Affiliations:** 1 International Laboratory of Quantum Optoelectronics, National Research University Higher School of Economics, 16 Soyuza Pechatnikov, St Petersburg 190008, Russia; nataliakryzh@gmail.com (N.V.K.); moisei-90@mail.ru (E.I.M.); anndra@list.ru (A.S.D.); 2 Department of Electronic and Electrical Engineering, Faculty of Engineering Science, University College London, London WC1E 7JE, UK; mingchu.tang.11@ucl.ac.uk (M.T.); siming.chen@ucl.ac.uk (S.C.); huiyun.liu@ucl.ac.uk (H.L.); 3 Center for Physics of Nanoheterostructures, Ioffe Institute, 26 Polytechnicheskaya, St Petersburg 194021, Russia; Marina.Kulagina@mail.ioffe.ru; 4 Laboratory of Nanophotonics, St. Petersburg Academic University, 8/3 Khlopina, St Petersburg 194021, Russia; skadinskaya@bk.ru (S.A.K.); fedyazu@mail.ru (F.I.Z.); alex000090@gmail.com (A.M.M.); maximov.mikh@gmail.com (M.V.M.)

**Keywords:** semiconductor laser, microdisk laser, quantum dots, III–V on Si

## Abstract

An InAs/InGaAs quantum dot laser with a heterostructure epitaxially grown on a silicon substrate was used to fabricate injection microdisk lasers of different diameters (15–31 µm). A post-growth process includes photolithography and deep dry etching. No surface protection/passivation is applied. The microlasers are capable of operating heatsink-free in a continuous-wave regime at room and elevated temperatures. A record-low threshold current density of 0.36 kA/cm^2^ was achieved in 31 µm diameter microdisks operating uncooled. In microlasers with a diameter of 15 µm, the minimum threshold current density was found to be 0.68 kA/cm^2^. Thermal resistance of microdisk lasers monolithically grown on silicon agrees well with that of microdisks on GaAs substrates. The ageing test performed for microdisk lasers on silicon during 1000 h at a constant current revealed that the output power dropped by only ~9%. A preliminary estimate of the lifetime for quantum-dot (QD) microlasers on silicon (defined by a double drop of the power) is 83,000 h. Quantum dot microdisk lasers made of a heterostructure grown on GaAs were transferred onto a silicon wafer using indium bonding. Microlasers have a joint electrical contact over a residual *n*+ GaAs substrate, whereas their individual addressing is achieved by placing them down on a *p*-contact to separate contact pads. These microdisks hybridly integrated to silicon laser at room temperature in a continuous-wave mode. No effect of non-native substrate on device characteristics was found.

## 1. Introduction

In microdisk/microring lasers, high quality factors can be achieved even in resonators of a few micrometers in diameter [1,2]. Unlike other sorts of microlasers [3,4,5,6], the epitaxial structure is very similar to that of conventional edge-emitters, the post-growth process is relatively simple, and injection pumping is easy to implement. Strong carrier localization within individual quantum dots is favorable for the resistance of quantum-dot (QD) based light-emitters against non-radiative recombination at the resonator sidewalls [7]. These make QD-based microdisk/microring lasers prospective candidates for integration of light emitters with silicon-based electronic and photonic elements. In its turn, this can be useful in the implementation of optical communication and biodetection [8,9,10] systems. A significant progress has been demonstrated in QD structures monolithically grown on Si including the realization of edge-emitting lasers [11,12], and, very recently, microdisk/microring lasers [13,14,15,16]. 

At room temperature for a continuous-wave regime of operation, a low threshold of ∼0.6 mA (which corresponds to a threshold current density of about 0.9 kA/cm^2^) has been reported for a small QD microring laser with a radius of 5 μm and ring width of 3 μm grown on pre-patterned (V-grooved) Si substrate [13]. In [16], low threshold currents have been reported for QD microring lasers monolithically grown on a silicon substrate through an intermediate GaP buffer layer. For microlasers with an outer ring radius of 15 μm and a ring waveguide width of 4 μm, the threshold current of 1.8 and 2.7 mA was achieved in devices with an undoped and modulation *p*-doped active region, respectively. Taking into account the microring area, this corresponds to the threshold current density of 550 and 826 A/cm^2^, respectively.

In [15], a comparative analysis of the threshold characteristics of QD microring lasers grown on either a V-grooved Si substrate or on an exact (001) Si substrate with a GaP intermediate buffer layer has been presented. For the microlasers of relatively large diameters (from ~50 to 100 µm), low minimum threshold current densities ranging, respectively, from 0.42 to 0.3 kA/cm^2^ have been reported. In smaller mirolasers, 20–30 µm in diameter, the minimum value of the threshold current density was 0.6–0.61 kA/cm^2^. A similar value of 0.59 kA/cm^2^ has been achieved in QD microdisk lasers monolithically grown on Si substrate with 4^o^ miscut angle off (100) plane [14].

The smaller the size of the devices, the denser and more complicated an optoelectronic circuit can be realized with their usage. Moreover, the miniaturization of the laser is expected to improve its high-speed performance [17]. Devices of a smaller size are also helpful for single-mode lasing since the free spectral range between neighboring whispering gallery modes (WGMs) is inversely proportional to the perimeter of the resonator [18]. However, the abovementioned examples reveal that the threshold current density of QD microdisk/microring lasers directly grown on silicon substrates tends to increase in smaller devices rather than remaining constant. In particular, the threshold current density in such microlasers demonstrated to-date is noticeably inferior compared to broad-area QD lasers on silicon for which the continuous-wave lasing threshold as low as 62.5 A/cm^2^ has been reported [14]. All the above emphasizes a necessity to undertake efforts to improve the threshold characteristics of microlasers on silicon, especially those of smaller sizes.

Robust operation over a sufficiently long time is crucial for any semiconductor device. In case of III–V laser diodes on silicon, the reliability is an issue in view of their still poorer structural and optical quality compared to their counterparts grown on native substrates. In a reliability study of QD stripe lasers epitaxially grown on Ge/Si substrates [19], the time to failure of 4600 h was estimated for the best case. For that laser, after ~2100 h of constant current stress at 2 kA/cm^2^ and 30 °C, the output power dropped from its initial level by more than two times. In [12], a lifetime study on QD stripe lasers epitaxially grown directly on a silicon substrate allowed the authors to conclude that an extrapolated time to failure is about 10^5^ h, which is an order of magnitude lower than previously reported for QD stripe lasers grown on GaAs [20]. Reliability studies of microdisk/microring lasers on silicon, to our knowledge, have not been performed previously.

Epitaxial growth of high-quality III–V materials on silicon is still tricky as it is affected by lattice- and polarity-mismatch [21], as well as a significant difference in coefficients of thermal expansion [22]. Moreover, the realization of the standard complementary metal–oxide–semiconductor (CMOS) process on a silicon wafer with monolithically integrated III–V optoelectronic overlays is an issue. Hybrid integration of a III–V microlaser with silicon, which can be performed in various ways [23,24,25], is capable of providing full CMOS compatibility. In a majority of cases, InP-based materials are used, whereas reports on hybrid integration of QD-based microlasers on GaAs are rare [26]. Meanwhile an InAs/InGaAs/AlGaAs material system can offer advantageous over InP-based counterparts, which usually suffer from poor temperature stability [27]. For example, QD-based microdisks on GaAs showed continuous-wave (CW) operation above 100 °C [28]. 

In the present research, we report on the improvement of the threshold characteristics of microdisk quantum dot lasers made of an epitaxial heterostructure directly grown on a silicon substrate. An ageing test was done for the first time for QD microdisk lasers on silicon. We also study QD microdisks transferred onto a silicon wafer by indium bonding. To the best of our knowledge, this is the first report on injection-pumped quantum dot microdisk lasers hybridly integrated with silicon.

## 2. Monolithically Integrated III–V Quantum-Dot Microdisk Lasers

### 2.1. Epitaxial QD Heterostructure Grown on Si Substrate

**Figure 1:**
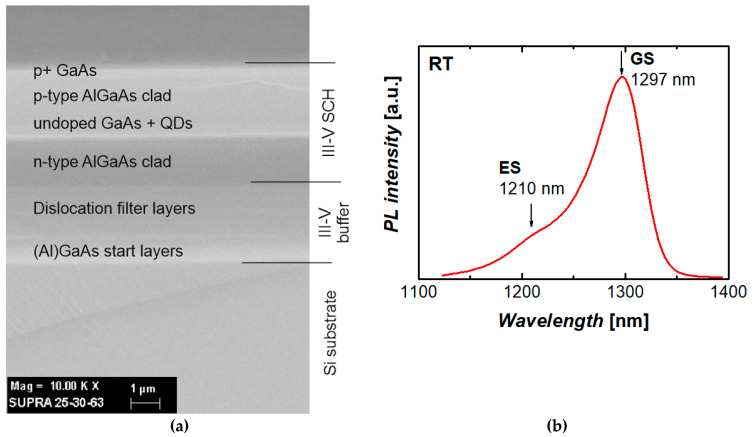
Scanning electron microscopy image of the cross-section of the III–V laser heterostructure grown on silicon (**a**) and representative photoluminescence spectrum of a quantum-dot (QD) test sample grown on silicon (**b**). GS and ES mean ground- and excited-state optical transitions, respectively.

A III–V epitaxial structure was grown by molecular beam epitaxy on a silicon (001) substrate. Oxide desorption was first performed by holding the silicon substrate at a temperature of 900 °C for 10 min. To facilitate a single-domain growth of III–V on silicon, the substrate was 4° misoriented toward the [011] plane. The growth starts with a 7 nm AlAs nucleation layer formed after oxide desorption by migration enhanced epitaxy using alternating Al and As_4_ flux at a low growth temperature of 350 °C and followed by deposition of an *n*+-doped buffer. In addition to GaAs layers, the buffer comprises a 50-period AlGaAs/GaAs superlattice of 200 nm and several InGaAs/GaAs dislocation filter layers [29] with a total thickness of 1.7 µm. By introducing a buffer of this sort, the density of threading dislocations propagating into the III–V layers from the III–V/Si interface can be significantly reduced below 10^6^ cm^‒2^ [30]. Planar interfaces and absence of micro-cracks were found by scanning electron microscopy (Supra 25, Zeiss, Switzerland) inspection of the structure’s cross-section (Figure 1a). 

A laser structure grown over the buffer represents a conventional separate-confinement heterostructure (SCH) with a ~0.43 µm thick undoped waveguide/active region sandwiched between 1.4 µm thick *n*- and *p*-type Al_0.4_Ga_0.6_As claddings. The active region was formed by seven planes of dot-in-well structures [31], each comprising 2 nm of In_0.18_Ga_0.82_As, 2.7 monolayers of InAs, and 6 nm of In_0.18_Ga_0.82_As and separated by 38.5 nm GaAs spacers. Capping a layer of quantum dots with a narrow-bandgap material results in a shift of the luminescence peak to longer wavelengths [32], e.g., 1.3 µm and beyond. The ground-state photoluminescence peak at 1297 nm is observed at room temperature with a full-width at half-maximum of ~60 nm for a laser test sample grown on Si. Note that the excited-state peak is well separated from the ground-state one.

Morphology and surface density of quantum dots gown on silicon, as revealed by transmission electron microscopy and atomic force microscopy are very comparable to those of QDs on a native GaAs substrate. A good QD uniformity is obtained with a density of ~3 × 10^10^ cm^‒2^ for an uncapped QD sample grown on Si; the typical dot size is ∼20 nm in diameter and ∼7 nm in height. Broad-area lasers (made of a similar QD epitaxial heterostructure grown on Si revealed a low threshold-current density of 62.5 A/cm^2^ at room temperature, operation up to 120 °C, and an extrapolated mean time to failure of over 10^5^ h [12]. 

### 2.2. Formation of Microdisk Laser Resonators and Experimental Details

**Figure 2:**
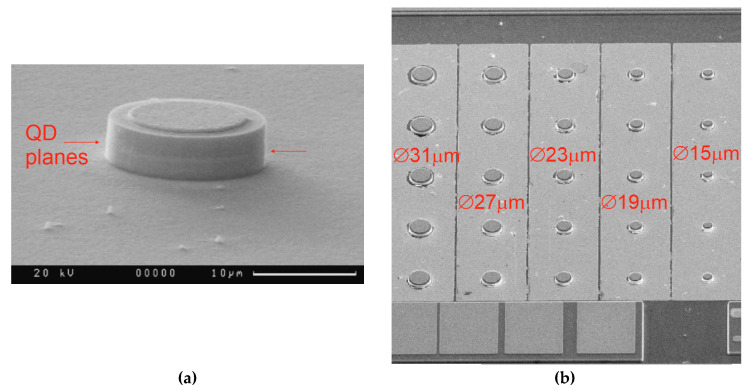
Scanning electron microscopy images of a microdisk with a diameter of 15 µm (**a**) and arrays of microdisks of different diameters (**b**) made on Si substrate.

Microdisk lasers on a silicon substrate were fabricated using the technique we previously developed with similar microlasers on GaAs [28,33]. Cylindrical mesas were formed by an inductively coupled BCl_3_/Ar plasma dry etching process using a photolithographically defined pattern. The etch mesa height is about 4.5 µm, i.e., the heterostructure was deep-etched through the active region (Figure 2a). Microdisks have a diameter varying from 15 to 31 µm with a step of 4 µm (Figure 2b). No passivation or sidewall coating was applied. The deviation of the side walls from verticality was better than 5 degrees. 

**Figure 3:**
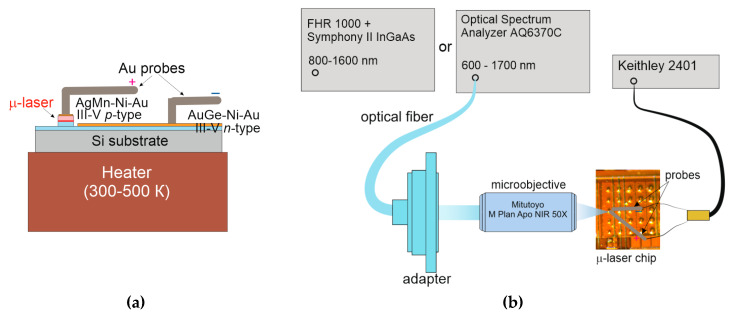
Microdisk electrical connection scheme (**a**) and sketch of optical measurement system (**b**).

Circular-shaped ohmic contacts to the *p*^+^-GaAs upper layer on top of the individual microdisks were made using Ag-Mn/Ni/Au metallization. The *p*-metal diameter was a few micrometers smaller than the mesa size. An *n*-type Au-Ge/Ni/Au ohmic contact was made common for a group of several microdisks of the same size. It was placed onto the etched surface of the underlying *n*^+^-GaAs layer between the microdisks. The annealing temperature was about 450 °C. Next, a chip comprising a plurality of microdisks was mounted onto a copper holder (Figure 3a), which temperature can be varied by a homemade heater in a wide range. When the heater was switched off, the ambient temperature during the characterization was about 26 °C.

The microdisks were electrically pumped using gold needle probes. Positioning was visualized using a visible-light camera. Direct current was controlled with a sourcemeter (Keithley 2401, Keithley Instruments, Solon, OH, USA). The current density was calculated as the injection current divided by the mesa area. Laser characteristics were evaluated using electroluminescence spectroscopy. Light emitting into free space by an individual microlaser was collected with an adjustable Mitutoyo M Plan Apo NIR objective with x50 magnification. Emission spectra were acquired with either a Yokogawa AQ6370C optical spectrum analyzer or a Horiba FHR 1000 monochromator equipped with a Horiba Symphony II InGaAs charge-coupled device (CCD) detector (Figure 3b). 

**Figure 4:**
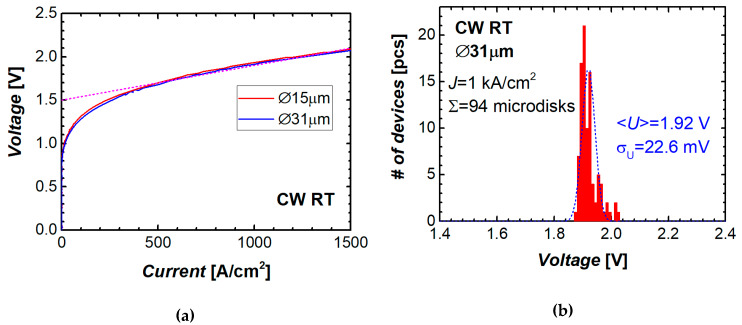
(**a**) Current–voltage curves of microdisk lasers of different diameters, dashed line represents the linear approximation; (**b**) bar diagram showing the distribution of diode voltage at constant current across 94 microdisks.

Current–voltage characteristics of two microdisk lasers of different diameters are presented in Figure 4a. The I–V curves presented in terms of ‘voltage against current density’ are nearly independent of the microdisk diameter. Starting from a current density of about 500 A/cm^2^, the I–V curve can be fitted by a linear function with an intersection (turn-on voltage) of 1.5 V and a slope (specific series resistance) of 4 × 10^‒4^ Ω·cm^2^. Compared to similar microdisks made of the heterostructures on *n*+ GaAs substrates [28,33], the series resistance of the studied microlasers on Si was found to be several times higher. 

Reasonable reproducibility of the fabrication process of QD microlasers on silicon was confirmed by narrow histograms of the parameter distributions. An example is shown in Figure 4b, where the data are depicted on diode voltage measured at a constant current density of 1 kA/cm^2^ among almost a hundred identical microdisks tested. The root mean square value of the deviation (RMSD) from the average voltage drop (1.92 V) is only 23 mV, i.e., 1.2%. As for the output power, the RMSD is about 20%.

### 2.3. Spectral Characteristics of Microdisks

**Figure 5:**
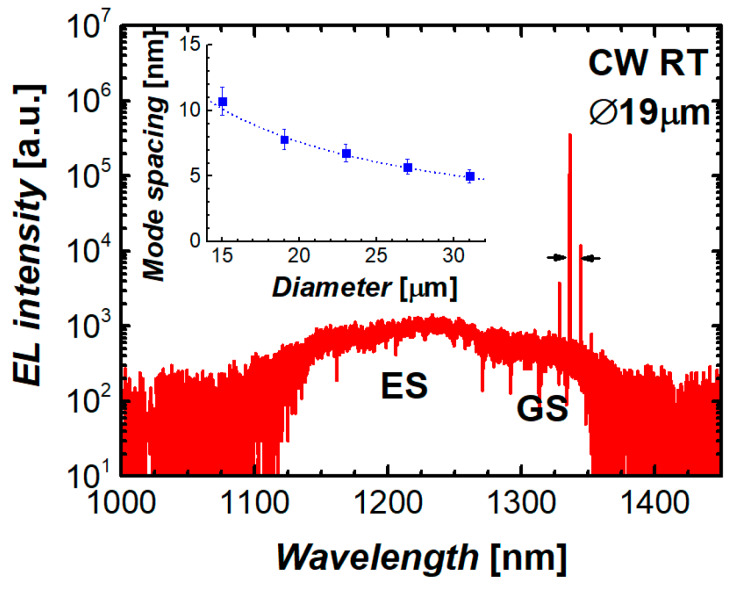
Representative emission spectrum of 19-µm microdisk laser (inset: mode interval vs microdisk diameter, dotted line: ~1/*D*).

Emission spectra of the microdisks under study comprise a series of narrow lines, which are attributed to WGMs of different azimuthal numbers, superimposed over a broad spontaneous emission of the quantum dots. An example of the emission spectrum of a 19 µm microdisk laser is presented in Figure 5. The WGM lines are located within the QD ground-state optical transition band well above 1.3 µm at room temperature. In the lasing regime, the intensity of the dominant mode is several orders of magnitude higher than the spontaneous-emission background. The free spectral range, i.e., the spacing between the nearest modes, is varied inversely with the microdisk diameter (inset to Figure 5), thereby confirming the WGM nature of the modes.

**Figure 6:**
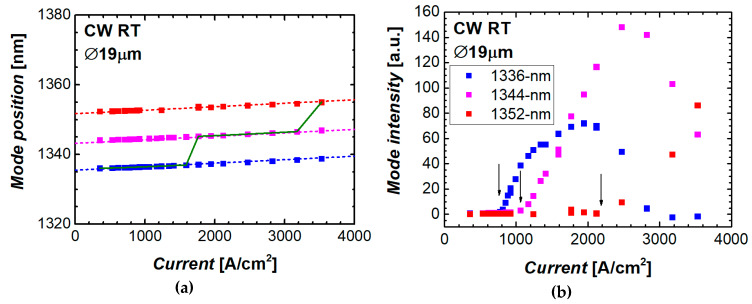
Spectral position (**a**) and integrated intensity (**b**) of the three most intense modes of the 19 µm microdisk laser against current density. Dotted lines in (**a**) correspond to the slope of 1 nm/(kA/cm^2^), solid line—spectral position of the most intense mode; arrows in (**b**) indicate the mode threshold.

As the level of the injection increases, a spectral position of the mode shifts to longer wavelengths with a rate of about 1 nm per each kA/cm^2^ (Figure 6a). Actual dependence of the peak position on the current density is more complicated than linear as it reflects the device self-heating caused by the Joule heat (Section 2.6). 

A number of modes, which can be simultaneously observed in a lasing spectrum of a microlaser, is usually varied from two to four. At that, their relative intensity significantly changes with increasing the current density (Figure 6b). For a given mode, the intensity initially increases rapidly with the current upon reaching the mode threshold, saturates, and after that declines until it is completely quenched. For the 1336 nm mode of the 19 µm microdisk, its threshold, saturation, and quenching happen at ~0.77, 1.9, and 2.8 kA/cm^2^, respectively. 

Saturation and extinction of the mode line is accompanied by the ignition of another, longer-wavelength mode, so that with an increase in the current density there is a gradual change of the dominant mode. For the example shown in Figure 6b, in addition to the 1336 nm mode (which starts at the lowest mode threshold), there are two other intense modes, 1344 nm and 1352 nm, with the mode threshold of 1.1 and 2.2 kA/cm^2^, respectively. The wavelength, which corresponds to the dominant mode, hops from its initial position at ~1336 nm to ~1344 nm at the current density of about 1.8 kA/cm^2^ and then does it again to ~1352 nm at 3.4 kA/cm^2^.

### 2.4. Threshold Current Density of Microdisk Lasers

**Figure 7:**
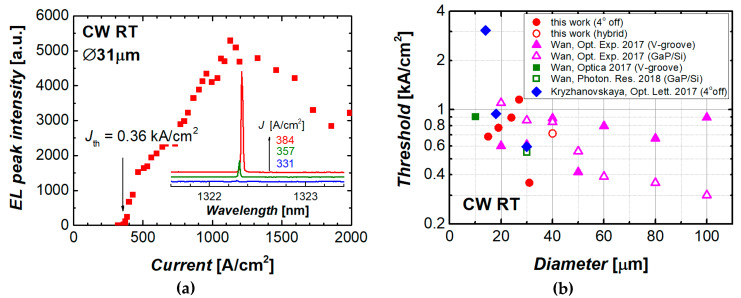
(**a**) Integrated intensity as function of current density for a 19-µm microdisk laser. Inset: evolution of emission spectrum near the threshold; (**b**) lowest threshold current density of our microdisk lasers on silicon against diameter (solid and open circles: monolithic and hybrid integration, respectively) in comparison with published results on monolithic GaAs-based QD microdisk/microring lasers on Si.

**Figure 8:**
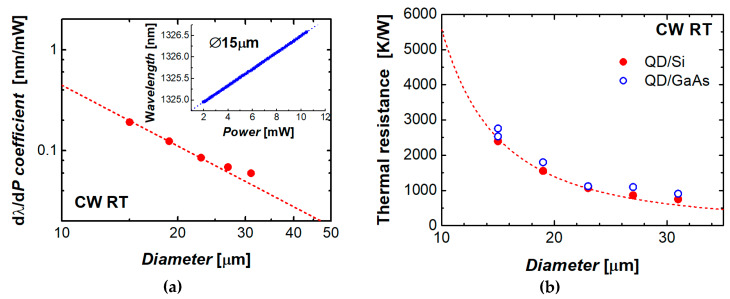
(**a**) Coefficient, which describes power-induced shift of the mode wavelength vs microdisk diameter. Inset: mode wavelength as function of consumed power for a 15 µm microlaser; (**b**) thermal resistance of QD microdisk lasers: solid circles—monolithic integration with Si, open circles—microlasers on GaAs. Dashed line: 5·10^‒3^ (K/W)·cm^2^.

The threshold current density of the microdisk laser was estimated from the light–current curve. We used the data of the WGM mode that has the lowest mode threshold among all modes of the given microlaser. The minimum threshold current density of 0.36 kA/cm^2^ was achieved in microlasers with a diameter of 31 µm (Figure 7a). To the best of our knowledge, this is the lowest threshold current density ever reported for WGM microlasers of comparable size made of a III–V heterostructure monolithically grown on silicon. This is more than one and a half times lower than our previous lowest value of 0.59 kA/cm^2^ reported for the microdisk laser of nearly the same size (30 µm in diameter) and similar structure (Figure 8). We attribute the progress to the improvement of the microdisk geometry that prevents mode leakage to the substrate and to the optimization of the etching process that allows for smoother sidewalls.

It is worth comparing the threshold current density obtained in the present research in microdisk lasers on silicon with our best result of 250 A/cm^2^ recently reported for quantum dot microdisk lasers on a GaAs substrate [34]. Those microlasers had a nearly identical diameter and were fabricated using the same post-growth process. Thus, the difference in the values of the threshold current density can be attributed to the difference in the crystal and optical quality of the laser structures grown on different substrates. 

In the studied III–V/Si microlasers of smaller diameters, the threshold current density is higher than in the 31 µm analog (Figure 7b). Meanwhile, the improvement is obvious compared to our previous results [14]. For example, for the 15 µm microdisks the previously achieved value of about 3 kA/cm^2^ was reduced down to ~0.7 kA/cm^2^. An absolute value of the threshold current was in this device of 1.2 mA. 

### 2.5. Thermal Resistancе of Microdisk Lasers and Lasing at Elevated Temperatures

**Figure 9:**
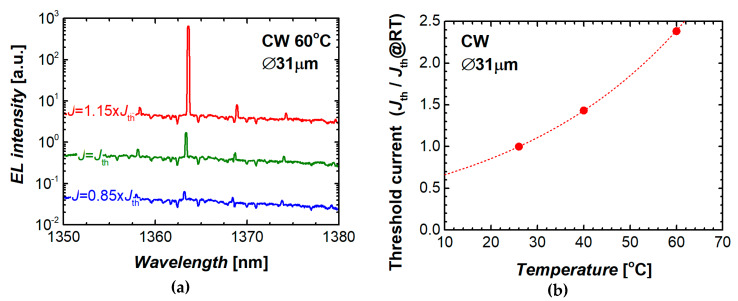
Series of emission spectra taken at 60 °C near lasing threshold (**a**) and temperature variation of threshold current (**b**). Dashed curve corresponds to characteristic temperature 39 K.

By definition, the thermal resistance *R*_T_ is a ratio of a temperature increment d*T* of the device active region over the ambient temperature to the heat d*P* released by the device operating in the CW regime. In microlasers, the output power is typically much lower than the electrical power and, therefore, the Joule heat approximately equals the consumed power. The latter is simply a product of the bias voltage and injection current. The experimental data on the current-induced mode shift, as was presented in Figure 6a, were used to evaluate the mode wavelength against the electrical power consumed by the III–V/Si microdisk laser. An example is presented in the inset to Figure 9a. It was found that the wavelength of a given mode is red-shifted with a constant slope with increasing power. The coefficient (dλ/d*P*), which describes this dependence, was found to scale with the microdisk diameter as *D*^–2^ (Figure 8a).

Combining the (dλ/d*P*) and (dλ/d*T*) coefficients (the latter is about 0.08 nm/°C near room temperature), we evaluated the microlaser thermal resistance as *R*_T_ = (dλ/d*P*)/(dλ/d*T*). The results are shown in Figure 8b. For the sake of comparison, the data are also presented for QD-based microdisk lasers on GaAs substrate as published in [35]. The thermal resistance of both sorts of microlasers can be adequately expressed as an inverse function of the area the microlaser footprint using the same specific thermal resistance of about 5 × 10^–3^ (K/W)·cm^2^. This indicates that the series resistance is predominantly controlled by the heat flow through the epitaxial layers with insignificant effect of both the substrate and the microdisk perimeter.

The microdisk QD lasers under study are capable of lasing at elevated temperatures. Figure 9a shows an evolution of the emission spectra with injection current density measured near the lasing threshold. The microlaser operates in the CW regime at the stage temperature of 60 °C. The dominant mode position is centered at around 1362 nm. Temperature stability of the threshold current is expressed by the characteristic temperature of 39 K (Figure 9b). 

### 2.6. Ageing Test of Microdisk Lasers

**Figure 10:**
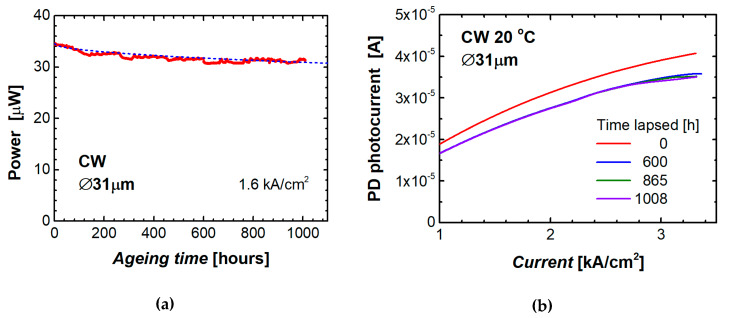
Ageing data for QD microdisk laser on silicon at a constant current density 1.6 kA/cm^2^; dashed curve corresponds to *P*(t)/*P*(0) = (1 – *a t*^m^) with *a* = 0.0031 and *m* = 0.51 (**a**). Photocurrent of monitor photodiode vs microlaser injection current measured during the test (**b**).

We performed for the first time a preliminary study of reliability of QD microdisk lasers on silicon. The ageing test was performed with a 31 µm microdisk laser operating without cooling or temperature stabilization with a constant CW current density of 1.6 kA/cm^2^, which corresponds to ~4 times the initial threshold current of that microlaser. The output power of the microdisk was periodically monitored with a minute interval (Figure 10a). After 1000 h of operation, the output power decreased by 9.2% from its initial level. 

Several times during the test, we recorded the light–current (L–I) curve of the microdisk laser over a wide range of injection current. To minimize possible variation of the light intensity caused by instability of the ambient temperature, these measurements were conducted with the laser placed on a heatsink stabilized at 20 °C by a Peltier thermoelectric cooler. The results are shown in Figure 10b. We found that after about 600 h of continuous operation of the microlaser, its L–I characteristic practically ceases to change. A small difference (about 2%) can only be found at high current densities (~3 kA/cm^2^), where the light intensity is close to its saturation level.

Temporal variation of the output power can be fitted as *P*(t) = *P*(0) (1 – *a t*^m^) with *a* = 0.0031 and *m* = 0.51. Previously, a similar equation (*I*_th_(t) = *I*_th_ (0) (1 + *a t*^m^) [36]) has been used to fit the threshold behavior over time for QD stripe lasers on silicon predicting the mean time to failure (MTTF) as MTTF = (1/*a*)^1/*m*^ [19]. Using this formula, we get for our microdisk laser the time to failure (defined by a two-fold decrease in power from its initial level) of 8.3·10^4^ h, which is comparable with the lifetime estimated for the stripe QD lasers made of the similar heterostructure grown on silicon [12]. We should emphasize it is a very preliminary estimation since the reliability study of a group of identical devices is required. 

## 3. Quantum Dot Microdisk Lasers Transferred onto Silicon Wafer

### 3.1. Sample Description

**Figure 11:**
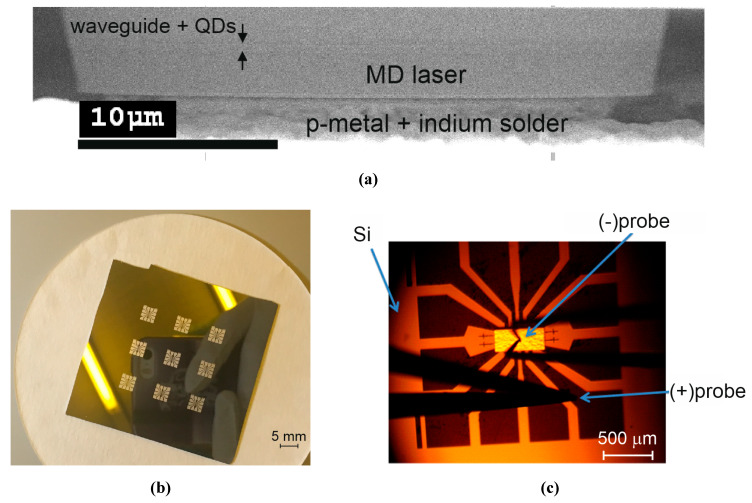
Contact pads formed on silicon substrate (**a**), scanning electron microscope image of the microdisk soldered onto Si substrate (**b**), electrical probes put on microdisk chip and contact pad (**c**)**.**

A heterostructure was grown by molecular-beam epitaxy on an *n*+-doped GaAs(100) substrate. It represents a conventional separate confinement laser structure with a GaAs waveguiding layer sandwiched between *n*-type and *p*-type Al_0.25_Ga_0.75_As claddings. The active region comprising 10 planes of InAs/In_0.15_Ga_0.85_As quantum dots deposited at a lowered temperature of about 510 °C was inserted into the middle of the waveguide with a thickness of ~0.44 µm (Figure 11a). Microdisk resonators were formed using plasma etching of photolithographically defined circular mesas. In the present research, we focus on the results obtained for mesas with a diameter of 40 µm. The etching was performed through the active region. No sidewall passivation was used.

Individual round-shaped contacts were made using Ag-Mn/Ni/Au metallization placed onto a *p*++-doped GaAs cap layer. The GaAs substrate was thinned down to ~100 µm, and a join *n*-type contact was put onto a back side of the substrate. After that, the wafer was cut into several chips each containing 12 microdisks. To ensure individual contacting of GaAs microlasers after their transfer onto the silicon substrate, contact pads were formed on the substrate surface using laser lithography, thermal evaporation, and the lift-off process. A combination of Cr/Au/In metals was used. Several groups of the contact pads were made (Figure 11b). Each group has a spider shape and is capable of providing a contact to each microdisk. Below, we focus on the results measured for microdisk lasers with a diameter of 40 µm.

The chip was then transferred onto the spider-shaped group of contact pads so that an individual microdisk was placed on a *p*-contact down onto an individual pad. Positioning was performed using a projection photolithograph. After that, the substrate was warmed up to ~200 °C followed by slow cooling. The chip was soldered by the indium thin film, which covers the Cr/Au contact pads (Figure 11a). The indium solder acts to hold the chip and simultaneously to provide electric contact to the microdisk. Two 50 µm-diameter gold-plated probes were used of which one was placed on the common contact on the back side of the chip and the second was put on the periphery of one of the contact pads (Figure 11c). The I–V curve demonstrates no impact of placing the disk on the non-native substrate. The series resistance normalized to the footprint area corresponds to a value of ~ 2.5 ×10^–4^ Ω·cm^2^.

Microlasers were tested at room temperature in a continuous wave regime. Neither cooler nor heatsinking was used. Emitted light was collected with an infrared objective, dispersed with a monochromator, and detected with an InGaAs CCD array.

### 3.2. Results on Hybridly Integrated Microdisks

**Figure 12:**
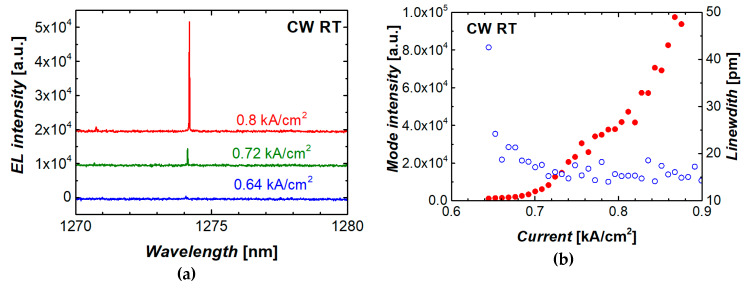
Evolution of emission spectrum near lasing threshold (**a**), mode integrated intensity (solid symbols) and linewidth (open symbols) against injection current (**b**) for microdisk laser transferred to silicon wafer.

Microlaser emission spectra taken at different injection currents are shown in Figure 12a. The dominant WGM line is at ~1274 nm, which is within the ground-state optical transition of the quantum dots used. Figure 12b shows the intensity of the dominant mode and its linewidth (full width at half maximum, FWHM) as functions of injection current density. The onset of lasing is confirmed by a rapid increase in intensity, which starts from the threshold of ~0.7 kA/cm^2^. Simultaneously, the FWHM drops down to 15 pm, which is the limit of spectral resolution of the optical system used. 

The threshold current density achieved corresponds to that of microdisk lasers before their transfer to the silicon wafer. Compared to the results on monolithic QD microlasers on silicon, the presently achieved threshold characteristics of the hybridly integrated QD microdisk are competitive (Figure 7b).

## 4. Conclusions

Progress made in epitaxial growth of high-quality quantum dot laser heterostructures grown on Si substrates and improvement of the post-growth process allowed us to improve the threshold characteristics of microdisk lasers on silicon. Compared to the previously presented results on the threshold current density of monolithic QD microdisk/microring lasers on silicon of comparable diameter, the threshold was improved by a factor of 1.5 down to 360 A/cm^2^ for 31 µm microdisks. In smaller microdisks (15 µm in diameter), the minimal threshold current density was found to be 680 A/cm^2^. We found that the silicon substrate has a marginal effect on thermal characteristics of microdisks laser, which value was found to be controlled by the III–V mesa diameter. The ageing test demonstrated that the temporal variation of the output power slows down with the time lapsed. As a result, after 1000 h the power decrement is only 9.2% from its initial value. The time to failure estimated for the microdisk laser is insignificantly shorter than that of the edge-emitting laser fabricated of a similar heterostructure on silicon.

CW lasing was demonstrated for the first time for injection-pumped InAs/InGaAs quantum dot microdisk lasers transferred onto a silicon wafer with the help of indium soldering. Microdisk lasers are individually addressed via separate contact pads. The current–voltage and thermal resistance of the hybridly integrated microlaser correspond to that of QD microdisk lasers on initial GaAs substrate. We believe, the method presented provides a simple technique for integration of ready optical micro-emitters with various electronic and optoelectronic systems embodied on substrates of different types.
